# Textile Waste-Derived Cobalt Nanoparticles Embedded in Active Carbon Fiber for Efficient Activation of Peroxymonosulfate to Remove Organic Pollutants

**DOI:** 10.3390/nano13192724

**Published:** 2023-10-08

**Authors:** Peiyuan Xiao, Ying Wang, Huanzheng Du, Zhiyong Yan, Bincheng Xu, Guangming Li

**Affiliations:** 1 State Key Laboratory of Pollution Control and Resources Reuse, College of Environmental Science and Engineering, Tongji University, Shanghai 200092, China; 1810308@tongji.edu.cn (P.X.); yingwang@tongji.edu.cn (Y.W.); yzyong77@163.com (Z.Y.); xubincheng012@163.com (B.X.); 2 UNEP-Tongji Institute of Environment for Sustainable Development, Tongji University, Shanghai 200092, China; dhz0403@126.com

**Keywords:** textile wastes, active carbon fiber, cobalt nanoparticles, embedded in, peroxymonosulfate activation, organic pollutant removal

## Abstract

Burning and dumping textile wastes have caused serious damage to the environment and are a huge waste of resources. In this work, cobalt nanoparticles embedded in active carbon fiber (Co/ACF) were prepared from bio-based fabric wastes, including cotton, flax and viscose. The obtained Co/ACF was applied as a catalyst for the heterogeneous activation of peroxymonosulfate (PMS) to remove bisphenol A (BPA) from an aqueous solution. The results showed that cotton-, flax- and viscose-derived Co/ACF all exhibited excellent performance for BPA degradation; over ~97.0% of BPA was removed within 8 min. The Co/ACF/PMS system exhibited a wide operating pH range, with a low consumption of the catalyst (0.1 g L^−1^) and PMS (0.14 g L^−1^). The high specific surface area (342 m^2^/g) and mesoporous structure of Co/ACF allowed the efficient adsorption of pollutants as well as provided more accessible active sites for PMS activation. This study provided an example of using textile wastes to produce a valuable and recyclable catalyst for environmental remediation.

## 1. Introduction

Worldwide textile consumption has increased from 47 million tons to 90 million tons in recent decades, and the growth rate will continuously expand with increases in population sizes and consumption levels [1]. At the same time, around 80% of textile wastes are disposed of via incineration and landfilling [2], creating huge environmental challenges, such as problems associated with energy and water consumption, scarcity of natural resources, and greenhouse gas emissions [3]. Corroborating on that fact, the latest EU reports revealed that textile wastes have contributed to more than 3% of greenhouse gas emissions in the EU [4]. As a result, the resource utilization of textile wastes has gradually aroused the interest of researchers. For example, Bansal et al. used textile wastes as a feedstock to produce biogas for energy recovery through chemical reactions [5]. Although the method is promising, the technique requires a complex pretreatment process to improve the yield and product quality, while energy production signifies a loss in the value of potentially recoverable cotton. Therefore, some researchers have attempted to produce valuable materials from textile wastes, including glucose, cellulose and regenerated fibers [6]. Lv et al. separated cellulose and nylon 6 using a dissolution process of ionic liquid 1-allyl-3-methylimidazolium from nylon/cotton fabric wastes [7], but they found that it was difficult to control the color and chemical structure of the product. Meanwhile, the generation of secondary pollution limits commercial applications [8]. Therefore, simple and eco-friendly methods should be investigated to realize high-value reutilization of textile wastes [9].

As a new type of activated carbon, activated carbon fiber (ACF) exhibits intriguing properties such as abundant surface functionality, uniform micropore volume, and large surface areas, which provide advantages for the rational design of functional materials as catalysts or adsorbents [10]. Currently, ACF is mainly synthetized from polyacrylonitrile (PAN), phenolic resin and pitch fibers by using non-renewable resources as the raw materials, making a significant contribution to lower the negative environmental impact [11,12]. Therefore, researchers are interested in exploring the synthesis of ACF from different biomass wastes, including textile wastes that consist of natural fibers and man-made fibers [13]. However, pre-treatments or modifications are needed to improve the physical and chemical properties of the resulting functional materials in the utilization of cellulose wastes [14]. As an example, most of the ACFs characterized in the literature mainly have microporous features rather than a mesoporous structure, limiting their applications for the adsorption of large molecules in aqueous solutions [15]. Silva et al. selected denim fabric waste to synthesize mesoporous ACF with the use of phosphoric acid as an action agent; the maximum adsorption capacity of ACF for Remazol Brilliant Blue R was 292 mg g^−1^, which was higher than that of pure carbon fiber without a pretreatment [16]. Metal element doping is another method used to modify surface properties and has advantages such as reduced equipment corrosion and environmental pollution due to the volatility and corrosiveness of the chemical by-products when compared to other activation methods [17]. Sun et al. prepared porous carbon adsorbents from waste cellulose fibers using ZnO as an activating reagent, which exhibited a superior removal ability of methyl orange (337.8 mg g^−1^) from an aqueous solution [18]. Nonetheless, the metal groups are often washed away by solvents such as inorganic acids in the process of material synthesis, which is not consistent with the purpose of green chemistry. Therefore, it is sensible to develop the application properties of the above metal species, rather than using them only as a sacrificial template to improve the performance of carbon fibers. 

At the same time, bio-based ACF has exhibited an advantage as a remarkable metal catalyst support owing to its strong structural durability, large surface area with microporous structure, and enhanced stability during the reaction process. Attempts have been made to explore the application potential of composite catalysts using ACFs as substrates in different fields, such as photocatalysis degradation of pollutants electrocatalytic hydrogen evolution reaction and hydrogen generation from an alkaline NaBH_4_ solution [19,20,21]. As one of the advanced oxidation technologies, peroxymonosulfate (PMS) has attracted extensive attention due to its high oxidation potential (2.5–3.1 V), long sulfate-free radical (SO_4_^•−^) life (30–40 μs), wide operating pH range and lack of sludge generation [22,23,24]. Generally speaking, SO_4_^•−^ is formed during electron transfer via multiple PMS activations, such as heat, UV irradiation and transition metal catalysis. Among them, the transition-metal (Co, Fe and Cu) activation method has been widely studied due to its low energy cost and simple reactor structure [6,25,26,27], while Co ion is considered to be the best choice compared to other metal ions due to its high redox potential (Co^3+^/Co^2+^; 1.82 V) [28]. Nevertheless, the possible toxicity and carcinogenicity of Co^2+^ have attracted attention. Therefore, cobalt-based heterogeneous catalysis is becoming a popular research topic. Recent studies have reported that dispersing Co particles on various carbon nanomaterials, such as graphene, porous carbon and multiwalled carbon nanotubes, can overcome the problems of low activity and stability, but the complex synthesis process and the high price limit the wide application of these methods. Meanwhile, ACF’s porous structure and rich functional group make it a promising support that can efficiently immobilize Co particles [29,30]. Co groups can also be used to modify the surface properties of ACF, which is prepared from different biomass wastes [31]. However, Co species loaded on ACF that is derived from actual textile wastes as an activator of PMS in handling organic pollutants has been rarely reported. 

The main objective of our work is to develop a cost-effective method for recycling high-valuable bio-based textile wastes and fabricating novel nanocomposites for environmental catalysis. A one-pot hydrothermal approach was utilized to synthesize activated carbon-fiber-supported cobalt nanoparticles (denoted as Co/ACF) by using bio-based shirt wastes (cotton, flax and viscose) as the raw materials. Specifically, cotton-derived activated carbon-fiber-supported cobalt nanoparticles (denoted as Co/CCF) were characterized in detail and applied for the removal of BPA via PMS activation. The possible catalytic mechanism of the Co/CCF/PMS system was also investigated. Moreover, flax shirt wastes and viscose shirt wastes prepared from the same strategy also presented remarkable catalytic ability for BPA removal. This study may provide a new avenue toward the efficient utilization of textile wastes for the production of valuable catalysts.

## 2. Materials and Methods

### 2.1. Chemicals and Materials

Cotton, flax and viscose shirt wastes were obtained from the Tianqiang municipal waste sorting center in Shanghai. Cobalt nitrate hexahydrate (Co(NO_3_)_2_·6H_2_O), bisphenol A (BPA) and potassium peroxymonosulfate (Oxone, 2KHSO_5_·KHSO_4_·K_2_SO_4_) were purchased from Aladdin Chemistry Co., Ltd., Shanghai, China. Methanol (MeOH, HPLC), ethanol (EtOH), tert-butanol (TBA) and potassium iodide (KI) were supplied by Sinopharm Chemical Reagent Co., Ltd., Tianjin, China. All reagents were used directly without further purification. The aqueous solution used in the experiments was prepared with deionized water (18.2 MΩ/cm) produced from Milli-Q System. 

### 2.2. Synthesis of Co/ACF and BPA Removal Experiments 

Co/CCF composites were prepared using a one-pot pyrolysis approach. In a typical way, the cotton shirt waste was shredded into small pieces with a size of 4 × 4 cm^2^, which were ultrasonically cleaned in a detergent solution for 1 h to remove oil stains that might exist on the surface. Then, the cotton pieces were rinsed several times using DI water and dried at 60 °C in a drying oven. Afterward, 0.05 g, 0.1 g, 0.2 g or 0.4 g of Co(NO_3_)_2_·6H_2_O (Co source) was dissolved in 15 mL of the aqueous solution, and the washed cotton waste pieces were immersed in the solution overnight to bind Co^2+^, followed by drying at 60 °C in a vacuum oven to obtain pink cotton pieces. The above precursor was carbonized at 800 °C for 4 h with a ramping rate of 10 °C/min under N_2_ protection. After naturally dropping to room temperature, it was taken out and grounded finely. Finally, the obtained samples were recorded as Co/CCF_0.05_, Co/CCF_0.1_, Co/CCF_0.2_ and Co/CCF_0.4_, respectively. As a control, Co(NO_3_)_2_·6H_2_O and the cotton waste pieces were separately thermally annealed under the same conditions, and the obtained samples were recorded as Co_3_O_4_ and pure CCF, respectively. Co/FCF_0.2_ and VCF_0.2_ composites were also prepared using the same method but with flax shirt waste and viscose shirt waste instead of cotton shirt waste.

In a typical test, the catalyst (0.1 g L^−1^) was dispersed in the BPA solution (50 mL, 40 mg L^−1^) with stirring for 60 min to achieve adsorption–desorption equilibrium between the BPA and the catalyst, and then, PMS (0.14 g L^−1^) was added to the mixture to initiate the reaction without any pH adjustment. At the desired intervals, 0.5 mL of the solution was collected and filtered using a syringe and a 0.45 μm water-phase needle filter film, which was mixed with 0.5 mL of MeOH to further quench the self-decomposition oxidation in the chromatographic vial for HPLC analysis. The samples were analyzed using a high-performance liquid chromatograph (HPLC, Shimadzu LC-20AT, Tokyo, Japan) equipped with a C18 column (4.6 mm × 250 mm × 5 μm) and a SPD-10A UV-visible detector set at the maximum absorption wavelength for BPA (273 nm). For the stability test, Co/ACF was collected after each run, repeatedly washed with deionized water and ethanol, and dried before the next recycle. 

### 2.3. Characterization and Analytical Methods

The crystal structure analysis was determined via powder X-ray diffraction (XRD) that was performed in a 2θ angle range of 10° to 80° using a Rigaku DMAX-2500 diffractometer with CuKα (λ = 1.54056 Å) as the radiation source. The surface element states were analyzed using an X-ray photoelectron spectroscope (XPS, K-Alpha, Thermo Scientific, Waltham, MA, USA) for producing narrow- and wide-scan spectra that was equipped with a non-monochromatic Al Ka X-ray source (1486.6 eV). The morphologies and microstructures of the prepared samples were obtained using a scanning electron microscope (SEM, Hitachi S-4800, Hitachi, Tokyo, Japan) and a transmission electron microscope (TEM, FEI Talos F200X, FEI, Hillsboro, OR, USA). The nitrogen adsorption–desorption isotherms were measured to analyze pore-size distribution and specific surface area using a physical adsorption analyzer (QuadraSorb SI, Quantachrome, Boynton Beach, FL, USA) working at −196 °C. Raman spectra were collected using a confocal Raman microscope (LabRAM HR 800, Horiba, Tokyo, Japan) equipped with an argon ion laser (514 nm). The reactive species were detected via electron spin resonance (ESR) experiments, which were performed using a Bruker A200 ESR spectrometer (Bruker, Preston, VIC, Australia) with DMPO as the spin trapper.

## 3. Results and Discussion

### 3.1. Structure and Morphology

**Figure 1:**
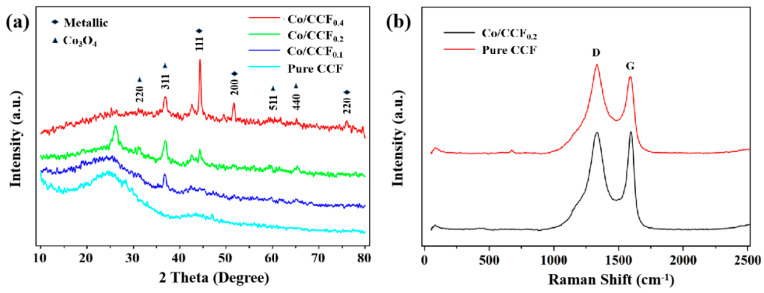
(**a**) XRD pattern and Raman spectra of CCFs doped with different amounts of Co. (**b**) Raman spectra of pure ACF and Co/ACF_0.2_.

The XRD patterns of Co/CCF well matched the crystalline structures of various shapes of cobalt-doped and porous activated carbon fibers (Figure 1a). The broad diffraction peaks around 20–30° correspond to the phase of graphitic carbons [32]. The peaks at 44.2°, 51.5° and 75.9° were assigned to the (111), (200) and (220) planes of zero-valence cubic-phase cobalt (Co^0^) [33]. Meanwhile, the peaks at 30.6°, 36.1°, 58.1° and 64.6° were due to the crystal planes of (220), (311), (511) and (440) of cubic-phase Co_3_O_4_, respectively [34]. It is shown that the intensity of individual CCF peak is gradually decreasing, which is accompanied by the increasing intensity of cubic-phase Co_3_O_4_ and Co^0^ peaks, from pure CCF to Co/CCF_0.4_. Previous research has reported that during carbonization process, Co^2+^ in Co(NO_3_)_2_·6H_2_O can be reduced to Co^0^, and the oxidization of Co takes place easily with oxygen groups on supported carbon at the surface of Co nanoparticles [35]. Therefore, we surmise that the existence of a multi-valence state of Co indicates an intense interaction between the Co element and the CCF, which is beneficial for immobilizing Co nanoparticles. In addition, when comparing 20%Co/CCF with 40%Co/CCF, the intensity of the Co_3_O_4_ characteristic peak presents significant difference, indicating Co species can be effectively anchored on the surface of the CCF.

The Raman spectra of pure ACF and Co/ACF_0.2_ are shown in Figure 1b. Two peaks are located at 1347 and 1589 cm^−1^, corresponding to the D band and G band. The intensity ratio of the D band to the G band (I_D_/I_G_) for Co/AC_0.2_ and ACF were determined to be 0.99 and 1.08, respectively. The decreased I_D_/I_G_ value reveals that the doping of CoNPs may lead to the production of more surface defects of Co/ACF_0.2_ when compared to pure ACF. These defects are known as important surface active sites for the adsorption of BPA and PMS activation [36].

**Figure 2:**
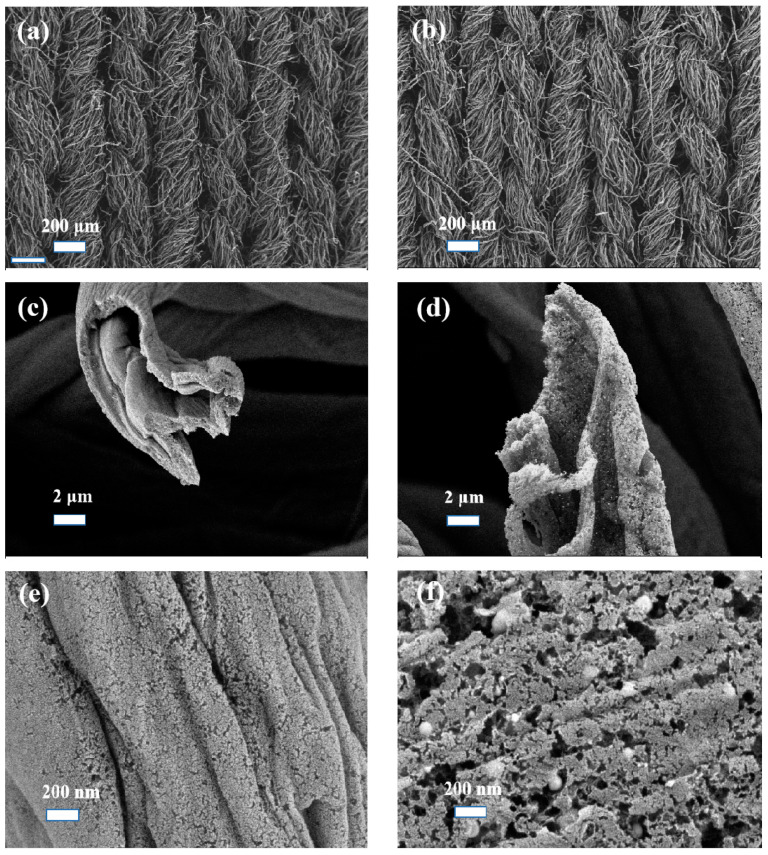
SEM images of (**a**,**c**,**e**) pure CCF and (**b**,**d**,**f**) Co/CCF_0.2_.

The SEM images show that Co/CCF maintains a hollow fibrous structure with a diameter of about 8 µm (Figure 2). This suggests that the doped CoNPs do not change the structure of the fixed interweaved fibers of cotton shirt waste. The surface of Co/CCF shows a mesoporous structure with CoNPs being evenly anchored on the channels and pores. This porous structure allows the efficient solute transfer from the solution to the solid active sites of the Co/CCF catalyst. As a comparation, Co/DCCF exhibits an irregular structure with abundant agglomerated nanoparticles on its surface (Figure S1). This structure may endow Co/DCCF with poorer catalytic activity due to the less exposed active sites. The TEM images confirm the evenly distribution of CoNPs in the active carbons with sizes in the range of 40 to 70 nm (Figure S2). From the HR-TEM images, the interplanar spacings of the lattice fringes of CoNPs were measured to be 0.20 and 0.24 nm, matching well with Co(111) and Co_3_O_4_ (331), respectively [37]. Comparing the SEM images shown in Figure 2e with Figure 2f, the significant difference indicates that the fibrous structure appears to be lost. This finding is in accordance with the results of the XRD patterns. 

The full-survey spectrum of Co/CCF confirms the existence of Co, O and C elements (Figure S3a). In the Co 2p XPS spectrum, the peaks at the binding energy of 779.1 and 793.9 eV can be attributed to Co 2p_3/2_ and Co 2p_1/2_ of Co^0^. The peaks at 782.9 and 796.2 eV correspond to the Co 2p_3/2_ and Co 2p_1/2_ states of Co^2+^, and the peaks at 780.1 and 794.7 eV can be ascribed to the Co 2p_3/2_ and Co 2p_1/2_ states of Co^3+^, respectively (Figure S3b) [38]. The O 1s spectrum of Co/CCF exhibits one more peak, which is attributed to the lattice oxygen of the Co_3_O_4_ phase when compared with pure CCF, again confirming the cooperation between CoNPs and CCF (Figure S3c,d).

### 3.2. Adsorption and Catalytic Degradation of BPA Using Co/CCF

**Figure 3:**
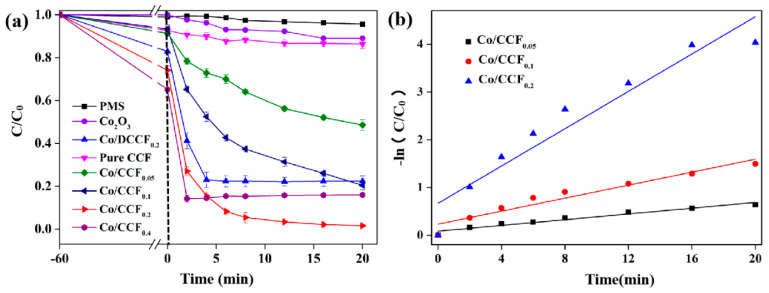
(**a**) The degradation curves and (**b**) their kinetic fit for the removal of BPA in different systems (initial BPA concentration = 40 mg L^−1^, dosage of catalysts = 0.1 g L^−1^, PMS = 0.14 g L^−1^).

The adsorption and catalytic activity of Co/CCF were evaluated via batch experiments. As shown in Figure 3a, in the absence of PMS, 9.6%, 11.2%, 15.6%, 26.8% and 36.6% of BPA could be adsorbed by pure CCF, Co/CCF_0.05_, Co/CCF_0.1_, Co/CCF_0.2_ and Co/CCF_0.4_, respectively. Additionally, the adsorption–desorption equilibrium was reached within 60 min. The concentration of BPA was almost unchanged in the presence of PMS alone after 60 min, indicating that PMS was inactive when used alone. Under the same reaction conditions of the Co/CCF/PMS system, ~11.2% of BPA was degraded by pure CCF within 20 min, while 56.3%, 81.6%, 99.9% and 83.5% of BPA was decomposed in the presence of Co/CCF_0.05_, Co/CCF_0.1_, Co/CCF_0.2_ and Co/CCF_0.4_, respectively. This suggested that Co/CCF_0.2_ exhibited the best performance among all the Co/CCF samples. In comparison, pure Co_3_O_4_ and Co/DCCF_0.2_ were also applied for the degradation of BPA via PMS activation. It was found that ~10.5% and ~81% of BPA was eliminated in the Co_3_O_4_/PMS system and the Co/DCCF/PMS system, respectively. These results indicated that Co/CCF showed superior degradation activity in BPA removal due to the synergistic effect of simultaneous adsorption and catalysis via the abundant pore channels and embedded CoNps. 

The plots of −ln(C/C_0_) vs. t are shown in Figure 3b. The good linearity between −ln(C/C_0_) and t suggests that the data are well fitted with the pseudo-first-order kinetic model [39]:

−ln(C/C_0_) = *k*t (1)

where C and C_0_ are the BPA concentrations before and after the catalytic reactions, and *k* is the constant of degradation kinetics. The obtained *k* value was 0.195 min^−1^ for the Co/CCF_0.2_/PMS system, which was ~6.3 times the *k* value for Co/CCF_0.05_ (0.031 min^−1^) and ~2.8 times that of Co/CCF_0.1_ (0.068 min^−1^), confirming the importance of Co active species in enhancing the catalytic degradation of BPA.

**Figure 4:**
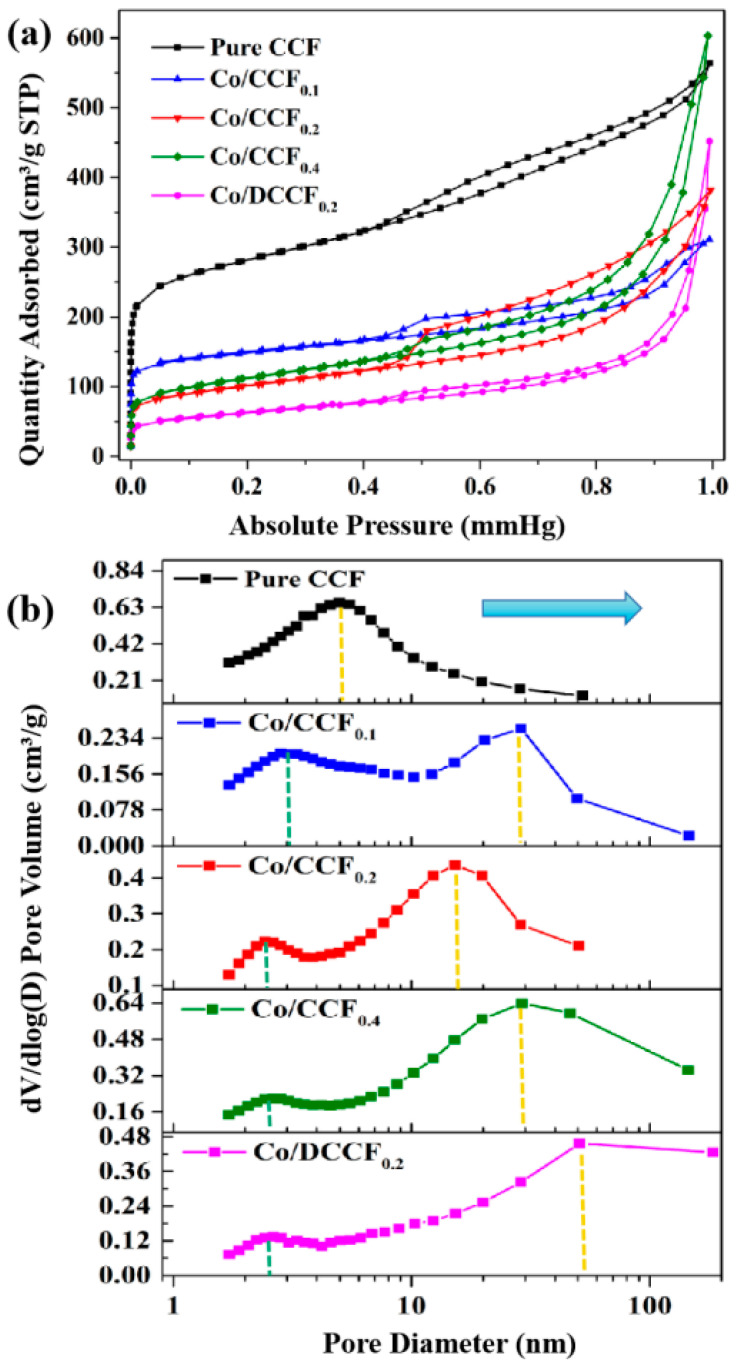
(**a**) N_2_ sorption isotherms and (**b**) corresponding pore-size distribution of CCF and DCCF doped with different amounts of Co.

The specific surface area, total pore volume and pore-size distribution of the various samples were investigated based on N_2_ adsorption–desorption isotherms. As shown in Figure 4a, all CCFs displayed type-IV isotherms, and a significant hysteresis loop appeared at P/P_0_ from 0.5 to 1.0, indicating that they all had a hierarchical porous structure with abundant micropores and mesopores. The specific surface area of Co/CCF_0.1_ (527 m^2^ g^−1^), Co/CCF_0.2_ (342 m^2^ g^−1^) and Co/CCF_0.3_ (375 m^2^ g^−1^) were all smaller than pure ACF (880 m^2^ g^−1^), indicating that CoNPs might have occupied some of the surfaces of CCF in the Co/CCF composites (Table S1). Interestingly, the ratio of micropores rose gradually with the weight increase in doped Co species, making it more convenient for BPA molecules to diffuse across the surface of the catalyst and interact [40]. The pore-size distribution of the various samples is shown in Figure 4b. In general, the mesopore size increased with an increase in the amount of CoNPs. Particularly, the most probable mesopore-size distributions of pure CCF, Co/CCF_0.2_ and Co/CCF_0.4_ were at 5 nm, 15.0 nm and 30 nm, respectively. The small pore size of pure CCF was a disadvantage for the migration of BPA molecules, while the too large pore size of Co/CCF_0.4_ weakened the interaction between CoNPs and ACF, resulting in lower stability and catalytic activity. This result was consistent with the degradation efficiency of bisphenol A shown in Figure 3. Meanwhile, the wide pore-size distribution of Co/DCCF_0.2_ was about 50 nm, which proved that the structure of the textile waste precursor played an important role in its excellent degradation efficiency.

### 3.3. Effect of pH and Reusability of Co/CCF

**Figure 5:**
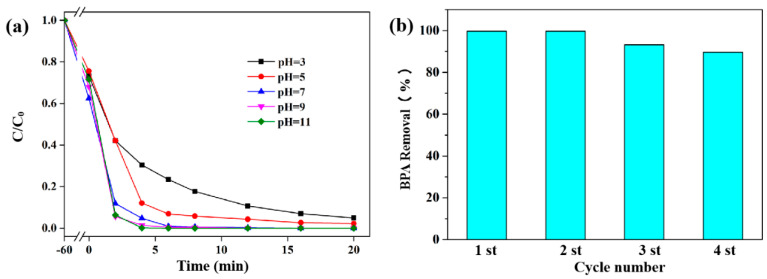
(**a**) Effect of pH and (**b**) recyclability of Co/CCF_0.2_ in the degradation of BPA after 20 min (initial BPA concentration = 40 mg L^−1^, dosage of catalyst = 0.1 g L^−1^, PMS = 0.14 g L^−1^).

The effect of the initial solution pH on BPA degradation was tested in a broad range of pH, ranging from 3.0 to 11.0. As shown in Figure 5a, when the initial solution pH varied from 7.0 to 11.0, BPA could be eliminated completely in the Co/CCF/PMS system within 8 min. At pH = 3, the degradation kinetic was much slower than that at a high solution pH, but BPA removal also reached 97% after 20 min. This might be because under an acidic condition, the strong hydrogen bonds between H^+^ and the O–O bonds of PMS inhibited the interaction between PMS and the Co sites of Co/CCF [38]. In addition, the formation of Co-OH complexes on the Co/CCF surface might impede the activation capacity of PMS and an increase in pH could weaken the hydrogen bonds [41]. Furthermore, it had been reported that the degradation of organic pollutants was inhibited at a low solution pH (pH < 5), owing to the scavenging effects of H^+^ ions for •OH and SO_4_^•−^ (Equations (2) and (3)).

OH• + H^+^ + e^−^ → H_2_O (2)

SO_4_^•−^ + H^+^ + e^−^ → HSO_4_^•−^ (3)

Reusability is of great importance for heterogeneous catalysts from an application point of view. As illustrated in Figure 5b, the degradation percentage of BPA in the Co/CCF/PMS system was maintained over 92% and 89.2% after three and four cycles, respectively. The slight decline in degradation capacity might be due to catalyst degradation. The excellent reusability might be attributed to the nanoconfined Co nanoparticles in the porous carbons of the CCF, which could be stabilized under harsh reaction conditions.

### 3.4. Identification of Active Species and Possible Degradation Mechanism

**Figure 6:**
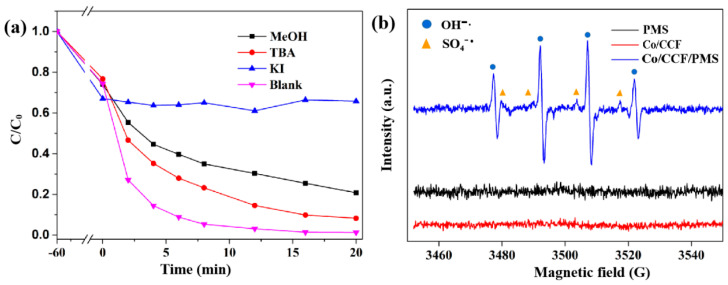
(**a**) Effect of radical scavengers on BPA degradation in the Co/CCF_0.2_/PMS system. Reaction conditions: [catalysts] = 0.1 g L^−1^, [PMS] = 0.14 g L^−1^, [BPA] = 40 mg L^−1^, and [scavenger] = 0.2 mol L^−1^; (**b**) EPR spectra of surface-adsorbed sulfate radical and hydroxyl radical.

Quench experiments were carried out to identify the reactive species for BPA degradation in the Co/CCF/PMS system. MeOH and TBA were used as radical scavengers for free SO_4_^•−^ and •OH in the aqueous solution (k (TBA, SO_4_^•−^) = 4–9.1 × 10^5^ M^−1^ s^−1^, k (EtOH, SO_4_^•−^) = 1.6–7.7 × 10^7^ M^−1^ s^−1^, k (TBA, •OH) = 3.8–7.6 × 10^8^ M^−1^ s^−1^, and k (EtOH, •OH) = 1.2–2.8 × 10^9^ M^−1^ s^−1^) [31]. Contrary to expectation, after an excessive amount of 200 mM of TBA was introduced into the Co/CCF systems, a negligible inhibition effect on BPA degradation was observed. Similarly, the degradation of BPA was suppressed by adding 200 mM of MeOH in the Co/CCF/PMS system, with the degradation percentage declining to 79.1%, indicating that SO_4_^•−^ and •OH radicals contributed to the degradation of BPA (Figure 6a). Such a special phenomenon usually occurs in surface-bound radical systems, where hydrophilic quenchers (MeOH and TBA) are difficult to accumulate on the catalyst surface to reduce the activity of the catalyst. KI was introduced into the system to determine the existence of surface-bound radicals [38]. Interestingly, the degradation of BPA was almost completely terminated by adding excess KI (10 mM) in the reaction system, indicating that surface-bound SO_4_^•−^ and •OH radicals played dominant roles in BPA oxidation.

To further analyze the reactive species in the Co/CCF/PMS system, we performed ESR analyses using 5,5-di-methyl-1-pyrroline N-oxide (DMPO) as a radical trapping agent. No signal was detected in the Co/CCF or PMS systems (Figure 6b), illustrating that PMS or Co/CCF alone could not produce reactive radicals. In the Co/CCF/PMS system, a strong signal of DMPO-SO_4_^•−^ and DMPO-•OH adduct could be observed. Based on these results, it can be concluded that surface-bounded •OH and SO_4_^•−^ radicals are responsible for BPA degradation.

**Scheme 1:**
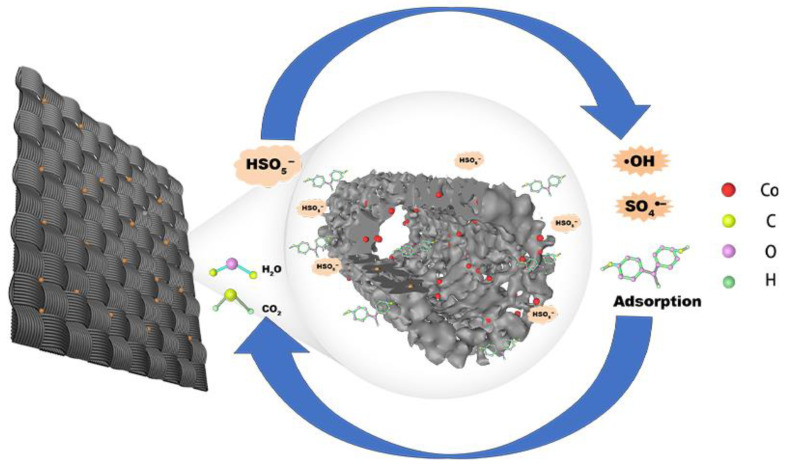
PMS activation mechanism by Co/CCF for BPA degradation.

A possible mechanism of BPA degradation via Co/CCF adsorption and catalytic activation of PMS is proposed. As shown in Scheme 1, Co/CCF can rapidly adsorb and enrich BPA from the solution to its surface. At the same time, Co/CCF can activate PMS to produce SO_4_^•−^ and •OH on its surface, and then oxidize and degrade the adsorbed BPA as well as release the adsorption sites. Such adsorption sites can further adsorb BPA in the solution and involve in a new cyclic adsorption process. The unique mesoporous structure allows the efficient adsorption of BPA on the surface of the catalyst, which makes the adsorbed BPA much closer to the cobalt catalytic centers. As a result, the adsorbed BPA can easily react with the surface-bounded radicals, and the synergistic effect between adsorption enrichment and catalytic degradation is responsible for the efficient degradation of BPA in the Co/CCF/PMS system.

The Co active sites play an important role in the activation of PMS. Firstly, Co^2+^ in the system can open the peroxide bond (-O-O-) of PMS to generate SO_4_^•−^ and •OH (Equations (4) and (5)). The activated Co^2+^ is transformed into Co^3+^ in the process. Since the redox potential of Co^3+^/Co^2+^ (1.808 V) is much higher than that of Co^2+^/Co^0^ (−0.277 V), the Co^3+^ generated in situ is easily reduced to Co^2+^ (Equation (6)) by wrapped CoNPs. At the same time, the generated Co^3+^ could also catalyze PMS to form SO_5_^•−^ with a lower reactivity than SO_4_^•−^ (Equation (7)). The generated SO_4_^•−^ and •OH could degrade BPA into intermediates and might even mineralize it into CO_2_ and H_2_O (Equation (8)). Moreover, the Co active sites are fixed on the Co/CCF surface via the coordination of Co^2+^/Co^3+^ with the oxygen-containing functional groups on the surface of CCF, which inhibits the dissolution of Co^2+^.

Co^2+^ + HSO_5_^−^ → SO_4_^2−^ + Co^3+^ + OH• (4)

Co^2+^ + HSO_5_^−^ → SO_4_^•−^ + Co_3_^+^ + OH^−^ (5)

Co^0^ + 2Co^3+^ → 3Co^2+^ (6)

Co^3+^ + HSO_5_^−^ → Co_2_^+^ + SO_5_^•−^ + H^+^ (7)

SO_4_^•−^/OH• + CIP → intermediates → CO_2_ + H_2_O (8)

### 3.5. Catalytic Activity of Co/FCF and Co/VCF

**Figure 7:**
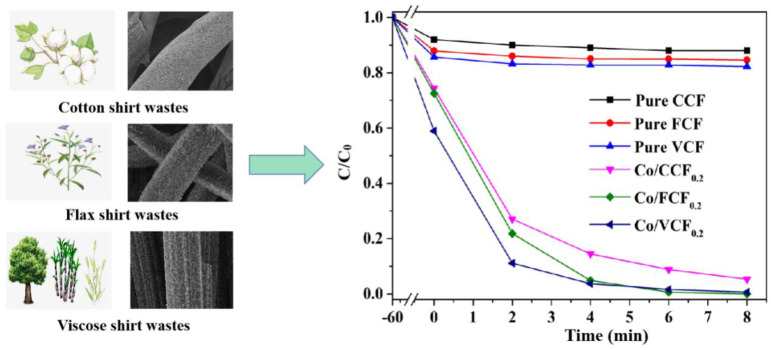
The degradation curves for the removal of BPA by carbon fibers derived from cotton, flax and viscose shirt wastes. Reaction conditions: [catalysts] = 0.1 g L^−1^, [PMS] = 0.14 g L^−1^, and [BPA] = 40 mg L^−1^.

Flax fiber and viscose fiber are widely used in textile products, which are derived from biomass materials, such as flax, trees, sugar cane and reeds. As a result, the performance of PMS activation via the use of Co/FCF_0.2_ and Co/VCF_0.2_ for BPA degradation was explored (Figure 7). With the addition of 20 wt% Co, the adsorption and degradation of all composites were significantly improved when compared to pure carbon fibers. Co/FCF_0.2_ and Co/VCF_0.2_ could adsorb 25.8% and 42.1% of BPA within 30 min, respectively, as well as achieve a complete degradation of BPA with PMS added in 8 min. These results show that transforming biomass textile wastes into Co/ACF_0.2_ as a PMS activator for highly efficient BPA degradation is promising. 

## 4. Conclusions

In summary, CoNPs embedded in ACF were fabricated using waste shirts (cotton fibers, flax fibers and viscose fibers) as the raw materials. Co/ACF exhibited a large specific surface area and a distinctive mesoporous structure with abundant active Co sites. These characters endowed Co/ACF with excellent catalytic performance to activate PMS for BPA degradation. In view of quenching tests and EPR analysis, surface-bound SO_4_^•−^ and •OH radicals were the main reactive species for the degradation of BPA. Additionally, the excellent adsorption properties of Co/ACF also facilitated the preconcentration of BPA on its surface and further boosted the in situ catalytic oxidation process. Co/ACF exhibited a wide operating pH range and good reusability. This work not only provided a brand-new idea for the recycling of various textile wastes but also encouraged further studies on the fabrication of novel catalysts for environmental remediation.

## Data Availability

The authors declare that they have no known competing financial interests or personal relationships that could have appeared to influence the work reported in this paper.

## Supplementary Materials

The following supporting information can be downloaded at https://www.mdpi.com/article/10.3390/nano13192724/s1, Figure S1: SEM images of Co/DCCF_0.2_ derived from degreasing cotton. Figure S2: (a,b) TEM and (b,c) STEM images of Co/CCF_0.2_; Figure S3: (a) XPS survey spectra of pure CCF and Co/CCF_0.2_; (b) high-resolution Co 2p spectrum and (d) O 1s spectrum of Co/CNF_0.2_; and (c) O 1s spectrum of pure CCF; Table S1: Porous textural properties of Co/CCF, Co/CCF_0.1_, Co/CCF_0.2_, Co/CCF_0.4_ and Co/DCCF_0.2_ composites measured based on nitrogen sorption.
