# Supplementary material for: Textile Waste-Derived Cobalt Nanoparticles Embedded in Active Carbon Fiber for Efficient Activation of Peroxymonosulfate to Remove Organic Pollutants
Source: Nanomaterials (Basel). 2023 Oct 8;13(19):2724. doi: 10.3390/nano13192724 (PMC10574149; doi:10.3390/nano13192724)
Supplement: Supplementary file 1 [file nanomaterials-13-02724-s001.zip › nanomaterials-2605337-supplementary.pdf]

Supplementary data

Textile wastes derived cobalt nanoparticles embedded in active carbon fiber for efficient activation of peroxymonosulfate to remove organic pollutants

Peiyuan Xiao[a,b], Ying Wang[a,b], Huanzheng Du[b], Zhiyong Yan[a], Bincheng Xu[a], , Guangming Li[a,b*]

[1]State Key Laboratory of Pollution Control and Resources Reuse, College of Environmental Science and Engineering, Tongji University, Shanghai, 200092, China

[2]UNEP-Tongji Institute of Environment for Sustainable Development, Tongji University, Shanghai, 200092, China

Corresponding author:

*Guangming Li, College of Environmental Science and Engineering, Tongji University, Shanghai, 200092, China

Tel./Fax: 86-13681662640, E-mail:ligm@tongji.edu.cn

Declarations of interest: none

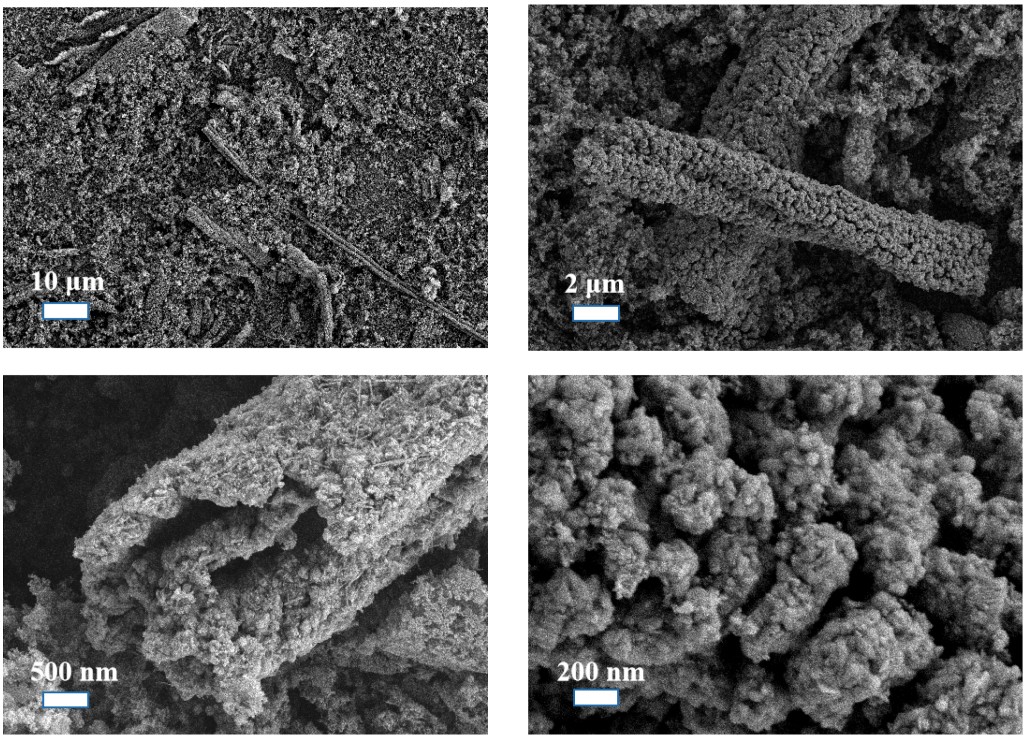

Fig. S1 SEM images of Co/$DCCF_{0.2}$ derived from degreasing cotton

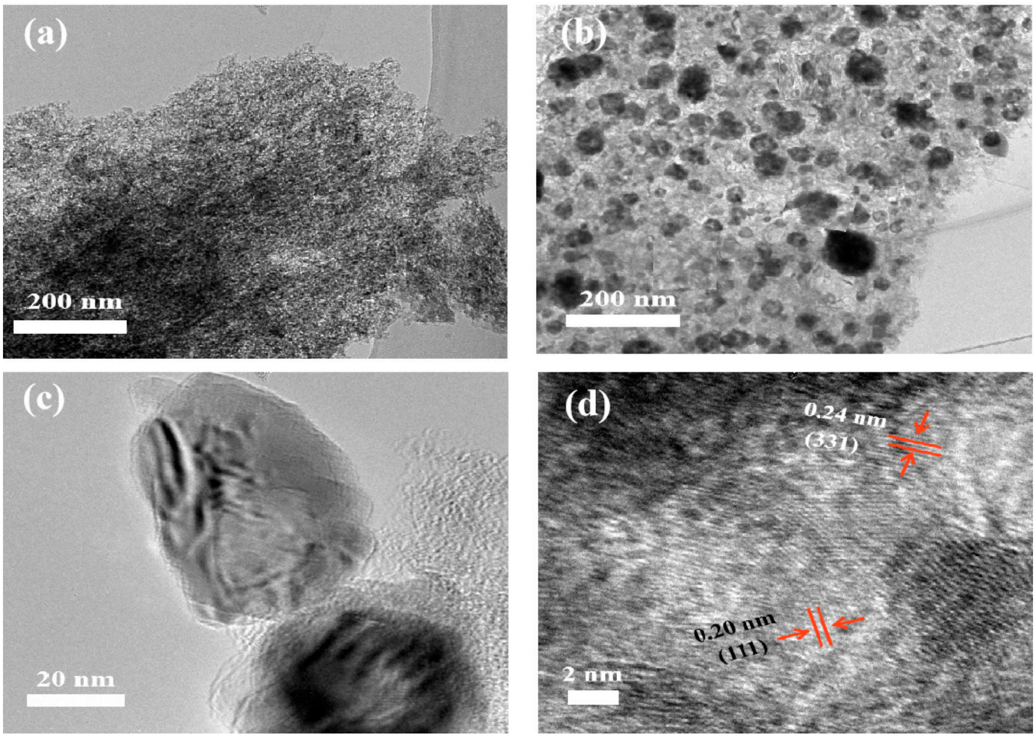

Fig. S2 (a,b) TEM and (b,c) STEM images of $Co/CCF_{0.2}$.

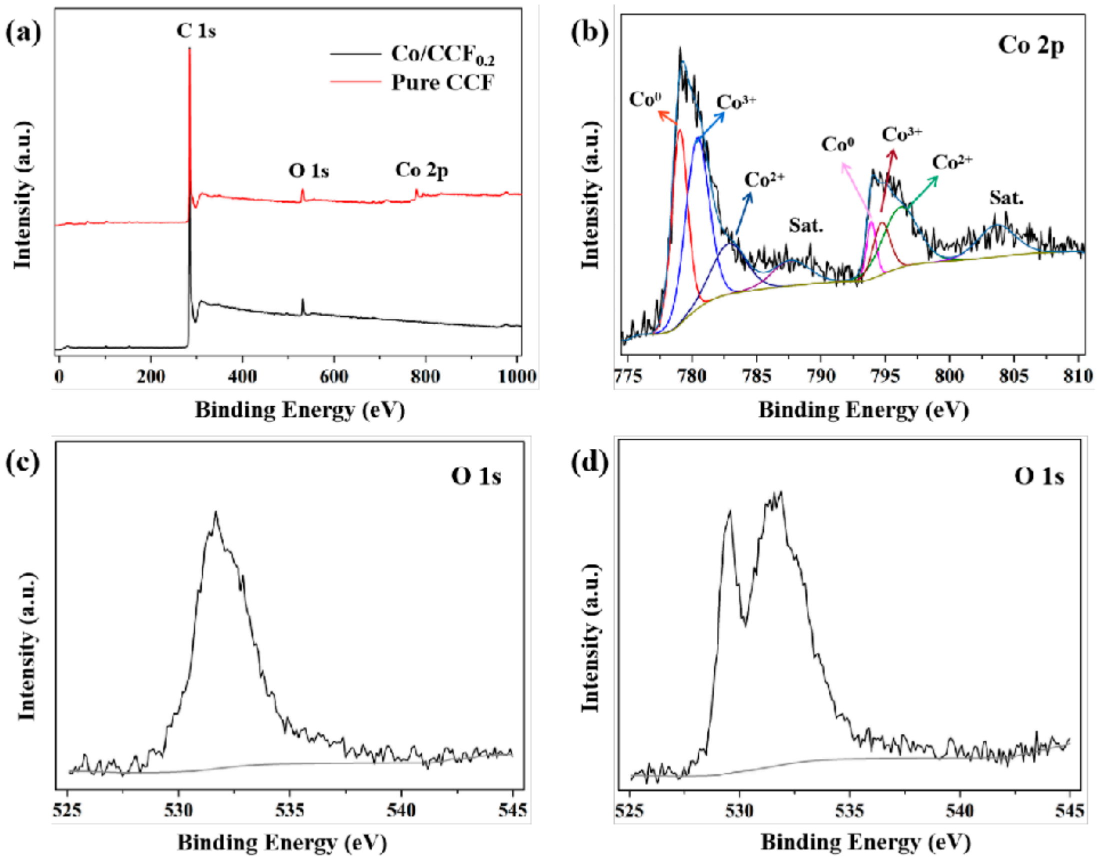

Fig. S3 (a) XPS survey spectra of pure CCF and Co/$CCF_{0.2}$; (b)High-resolution Co 2p spectrum and (d) O 1s spectrum of Co/$CNF_{0.2}$; (c) O 1s spectrum of pure CCF.

Table S1. Porous textural properties of Co/CCF, Co/$CCF_{0.1}$, Co/$CCF_{0.2}$, Co/$CCF_{0.4}$ and Co/$DCCF_{0.2}$ composites measured by nitrogen sorption

Sample	S_{BET} (m^2/g)	Total pore volume (cm^3/g)	Average pore size (nm)	Proportion of mesopores (%)
Co/CCF	880	0.807	5.24	27.75
Co/$CCF_{0.1}$	572	0.427	7.09	32.55
Co/$CCF_{0.2}$	342	0.461	8.35	9.32
Co/$CCF_{0.4}$	374	0.591	12.86	7.44
Co/$DCCF_{0.2}$	214	0.321	16.9	6.85
